# A Rare t(9;22;16)(q34;q11;q24) Translocation in Chronic Myeloid Leukemia for Which Imatinib Mesylate Was Effective: A Case Report

**DOI:** 10.4061/2011/592519

**Published:** 2011-07-05

**Authors:** Masahiro Manabe, Yumi Yoshii, Satoru Mukai, Erina Sakamoto, Hiroshi Kanashima, Takeshi Inoue, Hirofumi Teshima

**Affiliations:** ^1^Department of Hematology, Osaka City General Hospital, 2-13-22 Miyakojimahondori, Miyakojima-Ku, Osaka 534-0021, Japan; ^2^Department of Hematology, Osaka City University Medical School, 1-4-3 Asahimachi, Abeno-Ku, Osaka 545-8585, Japan; ^3^Department of Pathology, Osaka City General Hospital, 2-13-22 Miyakojimahondori, Miyakojima-Ku, Osaka 534-0021, Japan

## Abstract

The t(9;22)(q34;q11) translocation is found in about 90% of chronic myeloid leukemia (CML) patients. About 5–10% of CML patients have complex variant translocations involving a third chromosome in addition to chromosomes 9 and 22. Herein, we describe a CML-chronic phase male with a complex translocation involving chromosome 16, t(9;22;16)(q34;q11;q24). First, he was treated with interferon-alpha and intermittent hydroxyurea, but only a partial cytogenetic response was attained. Subsequently, the patient was treated with imatinib mesylate because of an additional chromosome abnormality, trisomy 8. A major molecular response was obtained after one year's imatinib therapy, and the follow-up chromosomal analysis performed 4 years and 3 months after the initiation of imatinib therapy displayed a normal karyotype of 46,XY.

## 1. Introduction

Chronic myeloid leukemia (CML) is characterized by the t(9;22)(q34;q11) translocation, in which the BCR gene at 22q11 is fused to the ABL gene at 9q34. The BCR/ABL fusion gene is thought to play a role in the leukemogenesis of CML and is a target of tyrosine kinase inhibitors, such as imatinib mesylate. 

About 5–10% of CML patients have complex variant translocations involving a third chromosome in addition to chromosomes 9 and 22. All chromosomes have been reported to act as the third chromosome, but breakpoint clustering at 1p36, 3p21, 5q13, 6p21, 9q22, 11q13, 12p13, 17p13, 17q21, 17q25, 19q13, 21q22, 22q12, and 22q13 has been reported [1, 2]. On the other hand, only a few cases involving chromosome 16 have been reported. Herein, we describe a CML-chronic phase male with a complex translocation involving chromosome 16, t(9;22;16)(q34;q11;q24), for which imatinib mesylate was effective, even after a new subclone, +8, appeared.

## 2. Case Report

**Figure 1:**
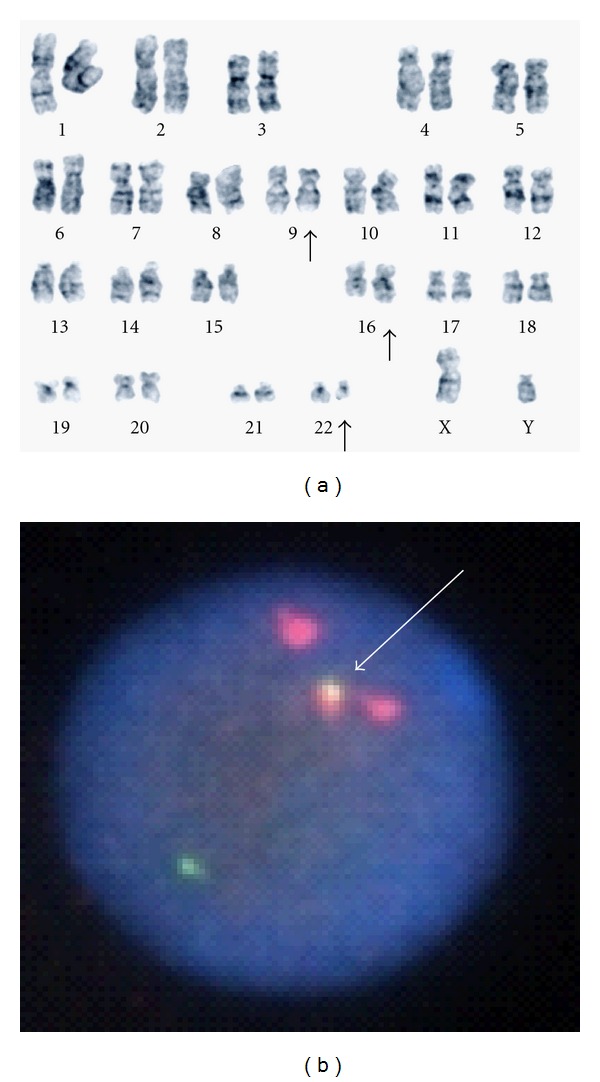
(a) G-band karyotype analysis performed at diagnosis revealed the following karyotype: 46,XY,t(9;22;16)(q34;q11;q24). (b) Dual-color FISH analysis performed using a BCR/ABL-specific probe (LSI BCR/ABL Dual-Color single translocation probe, Vysis, IL, US) demonstrated the presence of fusion signals (white arrow) in CML cells.

**Figure 2:**
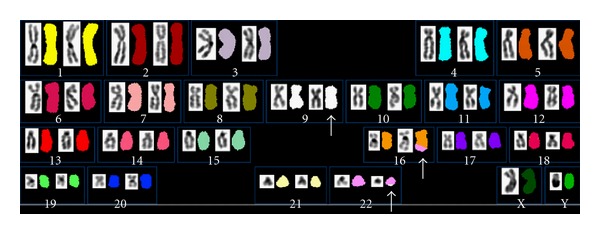
Multicolor FISH image (pseudocolor labeled) of metaphase spreads after spectrum-based classification.

A 55-year-old man was referred to us in November 2000 because of leukocytosis. No lymphadenopathy or hepatosplenomegaly was detected. Peripheral blood analysis showed a white blood cell count of 32.7 × 10^9^/L with 1% blasts, 0.5% promyelocytes, 5.5% myelocytes, 1% metamyelocytes, 64% neutrophils, 8% lymphocytes, 2% monocytes, 3.5% eosinophils, and 14.5% basophils. His hemoglobin level was 14.6 g/dL, and his platelet count was 339 × 10^9^/L. His serum level of lactate dehydrogenase was 423 U/L (reference range, 106–211 U/L). Bone marrow aspiration revealed less than 5% blasts, and karyotype analysis of his bone marrow cells showed the following karyotype: 46,XY,t(9;22;16)(q34;q11;q24) [20] (Figure 1(a)). In addition, fluorescent in situ hybridization showed BCR/ABL fusion signals in 92.2% cells, as shown in Figure 1(b). A diagnosis of chronic myeloid leukemia (chronic phase) was made. In addition, his prognostic Sokal score indicated that he was an intermediate-risk patient. He was treated with interferon-alpha and intermittent hydroxyurea from December 2000, and he achieved a partial cytogenetic response in February 2002: FISH showed fusion signals in 5.6% cells. However, cytogenetic surveillance on a quarterly basis confirmed that only a partial cytogenetic response had been attained, and a complete cytogenetic response was not achieved at any point during his treatment with this combination therapy. We proposed switching from interferon-based medication to imatinib mesylate, but he did not give his consent for this due to his economic situation. Follow-up cytogenetic analysis in November 2006 revealed the following additional abnormal karyotype: 46,XY,t(9;22;16)(q34;q11;q24) [3]/47,idem,+8 [2]/46,XY [4]. As shown in Figure 2, the complex translocation found by multicolor-FISH was consistent with the results of the G-banding analysis. Therefore, the patient was started on imatinib mesylate therapy (400 mg/day) and rapidly achieved a complete cytogenetic response (by January 2007). A major molecular response, estimated by the method described previously [3], was obtained after one year's imatinib therapy. The latest chromosomal analysis, which was performed 4 years and 3 months after the initiation of imatinib therapy, displayed a karyotype of 46,XY [20]. He still continues to display a major molecular response and is currently receiving imatinib therapy and undergoing quarterly cytogenetic evaluations.

## 3. Discussion

The t(9;22)(q34;q22) translocation is a characteristic chromosomal abnormality of CML, and in 5–10% cases, the BCR/ABL fusion gene is produced by a complex translocation. Several hundred CML cases with variant translocations have been reported, and the distribution of the breakpoints is clearly clustered around particular chromosomal bands such as 1p36, 3p21, 5q13, 6p21, 9q22, 11q13, 12p13, 17p13, 17q21, 17q25, 19q13, 21q22, 22q12, and 22q13 [1, 2]. To the best of our knowledge, chromosome 16 has only been reported to be involved in t(9;22)(q34;q22) variants in about ten cases [5–15]. However, most of these reports mainly focused on the chromosomal abnormality itself, and only a few reports mentioned the response to imatinib therapy; one showed hematological remission, three cases attained a complete cytogenetic response [8, 11, 15], and another case was not available. Although our case displayed a clonal chromosome abnormality (+8) in Ph+ cells at the start of the imatinib therapy, a major molecular response was achieved. Concerning the therapeutic response, in the preimatinib era, shorter survival was observed in variant Ph patients than in those with the classical Ph translocation, as deletions of the derivative chromosome 9 were found to occur at a much higher frequency in patients with variant translocations than in those with the classical t(9;22)(q34;q22) translocation [9]. However, it has been reported that patients harboring variant translocations have similar prognoses to those with classical t(9;22)(q34;q22) when treated with imatinib [14, 15]. On the other hand, although it was based on a small series of cases, it was reported that patients harboring complex variant translocations at diagnosis exhibit a poor clinical outcome because of their relative genomic instability compared to classical t(9;22) patients without variant translocations [4]. Hence, it seems that the prognostic impact of variant translocations has not been fully elucidated. In the present case, even after a new subclone, +8, had appeared, imatinib mesylate treatment achieved ample success. We think that further elucidation of the therapeutic outcome of imatinib therapy in CML cases involving variant translocations, including clonal evolution, is necessary.
